# The Emerging Role of Suppressors of Cytokine Signaling (SOCS) in the Development and Progression of Leukemia

**DOI:** 10.3390/cancers13164000

**Published:** 2021-08-08

**Authors:** Esra’a Keewan, Ksenia Matlawska-Wasowska

**Affiliations:** 1 Department of Pediatrics, Division of Hematology and Oncology, University of New Mexico Health Sciences Center, Albuquerque, NM 87131, USA; ekeewan@salud.unm.edu; 2 Comprehensive Cancer Center, University of New Mexico, Albuquerque, NM 87131, USA

**Keywords:** SOCS, leukemia, cytokine, cancer, JAK/STAT

## Abstract

**Simple Summary:**

The suppressors of cytokine signaling (SOCS) are known cytokine-inducible negative regulators of JAK/STAT and other cell signaling pathways. Deregulation of SOCS expression is linked to various tumor types and inflammatory diseases. While SOCS play a crucial role in the regulation of immune cell function, their roles in hematological malignancies have not been elucidated thus far. In this review, we summarize the current knowledge on the roles of SOCS in leukemia development and progression. We delineate the paradoxical activities of SOCS in different leukemia types and the regulatory mechanisms underlying SOCS deregulation in leukemia. Lastly, we discuss the possible implications of SOCS deregulation for leukemia diagnosis and prognosis. This paper provides new insights into the roles of SOCS in the pathobiology of leukemia and leukemia research.

**Abstract:**

Cytokines are pleiotropic signaling molecules that execute an essential role in cell-to-cell communication through binding to cell surface receptors. Receptor binding activates intracellular signaling cascades in the target cell that bring about a wide range of cellular responses, including induction of cell proliferation, migration, differentiation, and apoptosis. The Janus kinase and transducers and activators of transcription (JAK/STAT) signaling pathways are activated upon cytokines and growth factors binding with their corresponding receptors. The SOCS family of proteins has emerged as a key regulator of cytokine signaling, and SOCS insufficiency leads to constitutive activation of JAK/STAT signaling and oncogenic transformation. Dysregulation of SOCS expression is linked to various solid tumors with invasive properties. However, the roles of SOCS in hematological malignancies, such as leukemia, are less clear. In this review, we discuss the recent advances pertaining to SOCS dysregulation in leukemia development and progression. We also highlight the roles of specific SOCS in immune cells within the tumor microenvironment and their possible involvement in anti-tumor immunity. Finally, we discuss the epigenetic, genetic, and post-transcriptional modifications of SOCS genes during tumorigenesis, with an emphasis on leukemia.

## 1. Introduction

Leukemia is a common hematological malignancy that arises from aberrant proliferation and accumulation of transformed hematopoietic progenitors in the bone marrow, resulting in a profound inhibition of normal hematopoietic functions. It represents a heterogeneous group of myelogenous and/or lymphocytic malignancies further subdivided into acute and chronic types. In acute leukemias, such as acute lymphoblastic leukemia (ALL) and acute myeloid leukemia (AML), cell differentiation is arrested at earlier stages of cell development resulting in infiltration of the bone marrow with immature dysfunctional blasts. In contrast, in chronic leukemias, including chronic myeloid leukemia (CML) and chronic lymphocytic leukemia (CLL), cell differentiation is blocked at later stages of cell development leading to overproduction and accumulation of relatively mature and differentiated hematopoietic cells [1,2,3]. Leukemia results from multistep transformation processes in which leukemic cells acquire the ability to survive, proliferate, self-renew, evade apoptosis, and infiltrate various medullary, and extramedullary, tissues [1].

**Figure 1:**
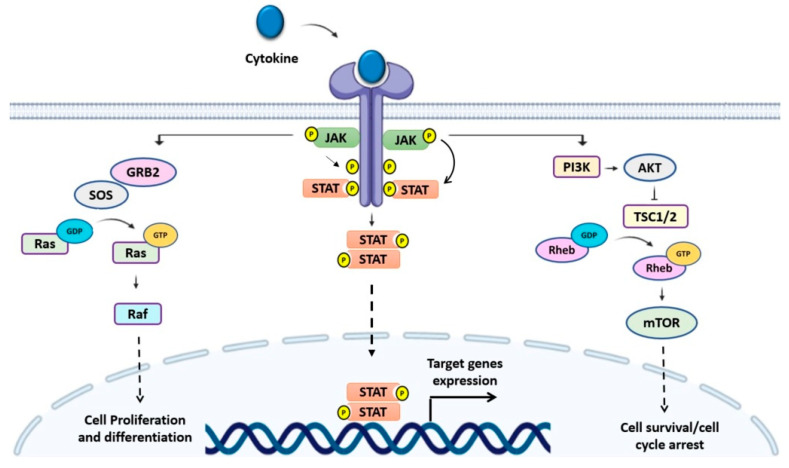
Schematic illustration of the Janus kinase signal transducer and activator of transcription (JAK/STAT) signaling cascade. The activation of JAKs subsequent cytokine binding results in the phosphorylation of STATs, which dissociate from the receptor, followed by dimerization and translocation to the nucleus to induce the transcription of the target genes. In addition to STATs, JAKs interact with various adaptor proteins, triggering several signaling cascades such as mitogen-activated protein kinase (MAPK), phosphatidylinositol-3-kinase (PI3K), a serine/threonine-protein kinase (AKT), and mammalian target of rapamycin (mTOR), which ultimately regulate cell proliferation, differentiation, cell survival, and cell cycle progression.

In normal hematopoiesis, cytokines play an essential role in regulating hematopoietic cell survival, proliferation, differentiation, and functional activation [2]. Pro-inflammatory cytokines are also crucially important for hematopoietic stem cell (HSC) maintenance, proliferation, and differentiation [4]. Building on this foundation, dysregulation of cytokine signaling has been associated with hematological malignancies, including various leukemia subtypes. The Janus kinase/transducers and activators of transcription (JAK/STAT) signaling pathways are activated upon cytokine binding with their corresponding receptors. In mammals, four JAKs (JAK1, JAK2, JAK3, and tyrosine kinase 2 [TYK2]) and seven STATs (STAT1, STAT2, STAT3, STAT4, STAT5A, STAT5B, and STAT6) proteins mediate signaling by more than 30 cytokine receptors. Ligand binding results in receptor dimerization and subsequent activation of JAKs. The activated JAKs phosphorylate specific tyrosine residues on the cytoplasmic tail of the receptor, creating docking sites for Src homology 2 (SH2)-containing proteins such as STATs, leading to the recruitment and subsequent phosphorylation of the target STATs. Once phosphorylated, the STATs disassociate from the receptor, followed by dimerization and translocation to the nucleus to initiate the transcription of the target genes [4,5]. The JAK/STAT signaling machinery interacts with numerous other cell signaling cascades such as mitogen-activated protein kinase (MAPK), phosphatidylinositol-3′-kinase (PI3K), a serine/threonine-protein kinase (AKT), and mammalian target of rapamycin (mTOR) [2,6] (Figure 1). In addition to JAKs, non-receptor tyrosine kinases such as SRC family kinases and the Abelson leukemia protein (Abl) directly activate STATs independent of ligand-receptor binding. The activation of non-receptor tyrosine kinases also leads to the activation of other downstream kinases such as p38, ERK, and JNK, which in turn induce STATs phosphorylation, and subsequent activation [2,7,8].

In normal cells, the activation of the JAK/STAT signaling pathways is transient and rapid due to tightly organized negative regulatory loops that ensure proper cellular response by preventing consecutive activation of the JAK/STATs and their downstream targets [9]. In leukemic cells, numerous genetic/epigenetic alterations trigger downregulation in negative regulators of signal transduction leading to constitutive activation of the JAK/STAT signaling pathways, which subsequently induces the activation of multiple cellular signaling cascades supporting malignant transformation [1,2].

Cytokine-induced JAK/STAT signaling is negatively regulated at several steps through distinct mechanisms, including protein tyrosine phosphatases (PTPs), protein inhibitors of activated STATs (PIAS), and suppressors of cytokine signaling (SOCS). These mechanisms tightly regulate the initiation, intensity, duration, and resolution of the JAK/STAT signaling cascades [10]. Genes encoding these negative regulators can be considered as tumor suppressor genes, as their inactivation significantly promotes malignant transformation, tumor cell proliferation and invasion, and cancer metastasis [2]. The SOCS family includes the pivotal negative regulators of JAK/STAT signaling and potent tumor suppressors. Mutations in the SOCS family genes are very rare and are found mainly in solid tumors and lymphomas [11,12,13,14]. However, silencing of *SOCS* expression is commonly seen in cancer and is associated with sustained activation of JAK/STAT signaling pathways, tumor aggressiveness, and poor prognosis [15,16].

On the contrary, upregulation of SOCS predominantly suppresses proliferation, invasion, and cancer metastasis leading to better clinical outcomes. However, a few contrasting reports demonstrated the link between SOCS upregulation and an inferior outcome [17,18]. Thus, the roles of SOCS in tumor development are likely dependent on tumor and cell types (Table 1). Interestingly, the abnormal expression of SOCS was also reported in immune cells found within the tumor microenvironment [9], suggesting the roles of SOCS in regulating cancer immunity.

Although a growing body of evidence points to the role of SOCS dysregulation in the development and progression of solid tumors, relatively little is known about whether and how SOCS regulates leukemia and other hematological malignancies. Here, we present SOCS family members as potential tumor suppressors or oncogene candidates and discuss their potential therapeutic, diagnostic, and prognostic implications in leukemia. We also summarize the roles of specific SOCS proteins in the tumor microenvironment and their potential role in the regulation of anti-tumor immunity. Lastly, we delineate epigenetic, genetic, and post-transcriptional modifications of SOCS gene expression and its effect on leukemia pathobiology.

## 2. SOCS Family Proteins

### 2.1. SOCS Structure and Function

**Figure 2:**
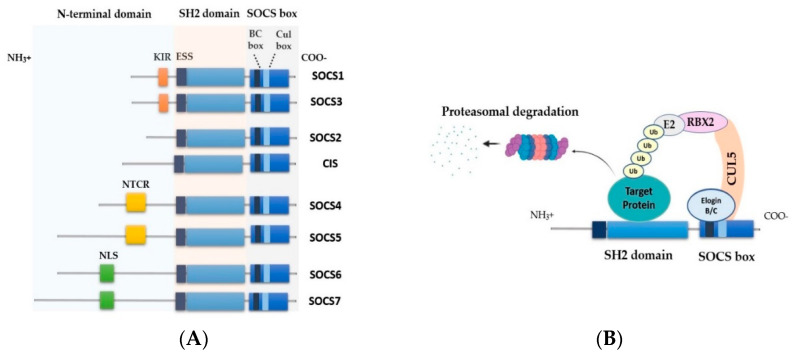
The structure and function of SOCS proteins. (**A**) The eight SOCS proteins contain a variable N-terminal domain, a central SH2 domain, an extended SH2 subdomain (ESS), and a C-terminal SOCS box. SOCS1 and SOCS3 have a kinase inhibitory region (KIR) that acts as a pseudosubstrate for JAKs and blocks their activity. SOCS4 and SOCS5 have N-terminal conserved regions (NTCR). SOCS6 and SOCS7 N-terminal regions contain putative nuclear localization signals (NLS). SOCS box is constituted of BC box and Cul box subdomains. (**B**) The SOCS box subdomains recruit Elongin B and C, Culin5 (CUL5), RING box protein-2 (RPX2), and other E3 ligase elements to induce ubiquitination of target proteins and their consequent proteasomal degradation.

The family of SOCS proteins plays a significant role in the regulation of intracellular signaling downstream of a wide range of receptors, including the cytokine receptors. This type of regulation is critical for the normal resolution of receptor signaling because it allows homeostasis to be restored by avoiding excessive cellular response. To date, eight SOCS family members have been identified in mammals, SOCS 1–7, and the cytokine-induced SH2 homology containing protein (CIS) [35]. All SOCS proteins share a similar architecture composed of a conserved central SH2 domain flanked by a well-conserved C-terminal SOCS box motif (40 residues) and an N-terminal region of varying length (33–385 residues) and limited homology (Figure 2A) [36,37]. SOCS 1–3 and CIS have a short N-terminal region (33–69 residues) compared to a much longer N-terminal region (270–385 residues) in SOCS 4–7.

Additionally, the N-terminal regions of SOCS 1 and SOCS3 are further discriminated by the presence of a 12-amino acid sequence, namely the kinase inhibitory region (KIR), which contains a conserved tyrosine residue that acts as a pseudosubstrate to block the active site that substrates would normally bind [38]. The N-terminal regions of SOCS 4 and SOCS5 have a highly conserved motif, known as the N-terminal conserved region (NTCR). The role of this region has not been identified thus far [39]. Further, both SOCS6 and SOCS7 have a putative nuclear localization signal (NLS) within the N-terminal region, which is required for their nuclear translocation and their ability to export/import other proteins into/out of the nucleus [40,41]. The SH2 domain of SOCS proteins serves as a substrate recognition domain; it specifically recognizes phosphotyrosine residues on the target proteins, imparting SOCS proteins with their target specificity [35]. The SH2 domain of SOCS1 binds specifically to the phosphotyrosine residues on activated JAKs, whereas CIS, SOCS2, and SOCS3 only bind to phosphotyrosine residues on the activated cytokine receptors [42]. Notably, the SH2 domain of SOCS proteins is elongated by an N-terminal extended SH2 subdomain (ESS) that provides further stability to the SH2 domain and enhances its substrate interaction [36,43].

A comparison of amino acid sequences across their SH2 domains shows partial homology between specific pairs of SOCS proteins; SOCS1/SOCS3 (share 37% of SH2 amino acids identity), SOCS2/CIS (share 45% of SH2 amino acids identity), SOCS4/SOCS5 (share 88% of SH2 amino acids identity), and SOCS6/SOCS7 (share 54% of SH2 amino acids identity) [44,45]. The highly conserved SOCS box domain recruits an E3 ubiquitin ligase complex, leading to the ubiquitination and subsequent degradation of the target proteins. Specifically, the SOCS box comprises two functional sub-domains, a BC box that mediates Elongin B/C binding and a Cul box that binds to the E3 ligase scaffold, Cullin-5 (CUL5). Subsequently, the resulting complex binds to the RING box protein-2 (RBX2), which ultimately recruits E2 ubiquitin-conjugating enzyme into the E3 ubiquitin ligase complex during ubiquitin transfer to a target substrate (Figure 2B) [46,47,48].

### 2.2. Control of Signaling by SOCS

**Figure 3:**
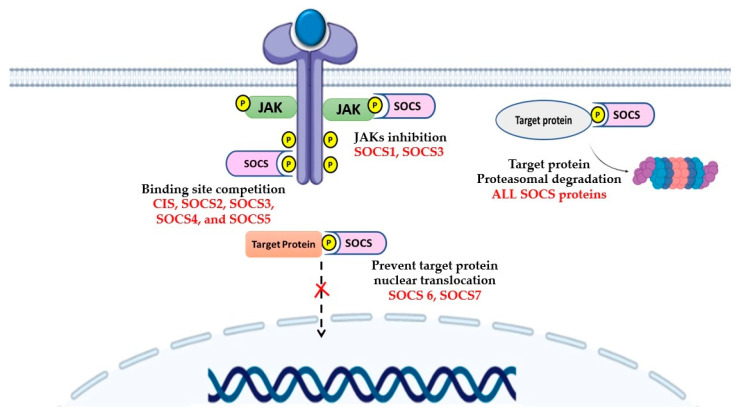
Mechanism of action of SOCS proteins. SOCSs negatively regulates intracellular signaling by several mechanisms: tagging the receptor or target protein for proteasomal degradation, blocking JAKs activity by acting as a pseudosubstrate, binding competition for the receptor phosphotyrosine that prevents the binding of other signaling molecules, and blocking nuclear translocation of signaling molecules.

SOCS proteins inhibit intracellular signaling by several mechanisms that vary between family members. All SOCS regulate intracellular signaling by recruiting proteasomal degradation machinery to their target proteins. SOCS recognize and bind to their target proteins through their SH2 domains, followed by the recruitment of E3 ubiquitin elements (Elongin B/C, CUL5, and RBX2) by their SOCS box resulting in ubiquitination and proteasomal degradation of the target proteins. Interestingly, the most potent SOCS family members, SOCS1 and SOCS3, have a low binding affinity for CUL5 and regulate signaling in a SOCS box-independent manner. Indeed, SOCS 1 and SOCS3 directly inhibit JAKs activity through their KIR domains. The KIR domain acts as a pseudosubstrate that binds to the phosphotyrosine residue of the JAKs, preventing these kinases from phosphorylating their substrates, including STATs. As another alternate mechanism, CIS, SOCS2, SOCS3, SOCS4, and SOCS5 inhibit the intracellular signaling by binding to phosphotyrosine residues on the receptor and by blocking the access of other SH2-containing signaling molecules. In addition, SOCS6 and SOCS7 suppress intracellular signaling by inhibiting the translocation of activated STATs to the nucleus, thereby preventing the transcription of their target genes [2] (Figure 3).

## 3. SOCS Proteins in Leukemia

Constitutive activation of the JAK/STAT signaling pathway is seen in leukemic patients with an unfavorable prognosis [49]. STAT1 and STAT5 are commonly activated in subsets of T-ALL and B-ALL [50,51], while STAT3 and STAT5 in AML [52,53,54]. Benekli et al. reported constitutive STAT3 activation in 44% of AML patients coincident with unfavorable clinical outcomes [54]. Many studies have linked the aberrant JAK/STAT signaling with leukemia initiation and progression [47,55,56,57]. More recently, the gain-of-function mutations in JAK1, JAK2, and JAK3 were identified in various leukemia subtypes [58,59,60,61]. Likewise, mutations in the IL-7 receptor and resulting constitutive activation of JAK1 were associated with pediatric T-ALL [62]. Altogether, a growing body of evidence underscores the importance of JAK/STAT activation in leukemogenesis. Thus, targeting aberrant JAK/STAT signaling has been considered an attractive strategy in targeting leukemic cells [2,56,57,63,64,65,66,67]. In addition to the profound role of genetic alterations in JAK/STAT activation, the aberrant activation of this pathway is also seen in leukemias that do not harbor mutations in JAK/STAT or IL7 receptor, suggesting that there are other mechanisms activating this pathway. While negative regulators function to terminate the activated signaling, their downregulation might prevent the shutting down of the activated pathways leading to the persistence and/or enhancement of aberrant signaling. Specifically, deregulation in negative regulatory loops could impair the receptor signal resolution leading to the constitutive activation of JAK/STATs. Thus, downregulation of negative regulators of signal transduction could potentiate the JAK/STAT signaling pathway when JAK/STAT mutations are present, or in some instances, this could be the primary driver underlying JAK-STAT pathway activation [68]. These observations point to the potential implication of SOCS family proteins, which are indeed negative regulators of JAK/STAT signaling, in the regulation of aberrant activation of JAK-STAT signaling. This section summarizes our current, still limited, understanding of the roles of various SOCS in leukemia pathobiology and clinical outcomes (Table 2).

### 3.1. SOCS1 and SOCS3

SOCS1 and SOCS3 have been the most extensively studied amongst the SOCS family members in relation to cancer and inflammation. SOCS1 and SOCS3 suppress cell growth and proliferation, and their expression is commonly downregulated in different types of cancer, including hematological malignancies such as ALL, CLL, and AML [37,71,84,85]. *SOCS1* overexpression impairs the transforming activity of several hematopoietic-specific oncogenes such as KIT, JAK2, and ABL [86]. Studies have shown that deletion of *SOCS1* drives the malignant transformation of fibroblasts. Accordingly, *SOCS1* overexpression significantly inhibited TEL-JAK2-mediated transformation and decreased the metastatic potential of *BCR-ABL* chimeras in Ba/F3 cells in vivo [86]. Similarly, SOCS1 inhibited TEL-JAK2 transformation of Ba/F3 cells in vitro and prolonged latency of TEL-JAK2-mediated disease in a murine bone marrow transplant model [87]. Growing evidence suggests the roles of epigenetic modifiers in the regulation of *SOCS* expression. *SOCS1* hypermethylation was found in 72% of primary AML cases and 52% malignant hematopoietic cell lines. These observations correlated with significant downregulation of *SOCS1* expression, suggesting a potential role of *SOCS1* silencing in promoting leukemogenesis [88]. Zhang et al. reported a significantly higher *SOCS1* methylation status in the initial treatment of relapsed/refractory AML corresponding to lower *SOCS1* mRNA and protein levels compared to remission and normal control samples [77]. Pharmacological demethylation of *SOCS1* in AML cell lines U937 and THP-1 increased the levels of SOCS1 mRNA coincident with decreased activation of JAK2, STAT3, and STAT5, decreased leukemic cell viability, and increased apoptosis. Similar results were obtained after transducing U937 and THP-1 cells with a plasmid expressing *SOCS1* [77]. In another study, ectopic expression of *SOCS1* inhibited cell growth of Jurkat T-ALL cells [88]. Consistently, lower *SOCS1* mRNA levels were also found in peripheral blood mononuclear cells of ALL patients compared to healthy control samples [89].

Genetic alterations of the *SOCS1* gene were observed in the Jurkat cell line and two primary AML samples. Sequence analyses of the *SOCS1* coding region in Jurkat cells revealed a one base pair deletion (G) at codon 164, leading to a frameshift mutation that replaces 40 amino acid residues at the C-terminal. A hemizygous missense mutation (C to T) at codon 198 resulting in the substitution of Ser for Pro was identified in two AML samples. However, the authors were not able to exclude the possibility that the missense variant represents a polymorphism [88].

Contrasting data on epigenetic regulation of *SOCS1* gene in CML have been reported. In studies by Hatirnaz et al., the *SOCS1* promoter was not methylated in the tested CML patient samples [90]. Another study using CML diagnostic samples identified *SOCS1* silencing by promoter hypermethylation. The methylation status was switched to unmethylated in molecular remission CML samples [91]. Others reported that *SOCS1* overexpression subverted cytogenetic response to IFN-α, and these observations were linked to poor prognosis [83]. Accordingly, a single nucleotide polymorphism (SNP) in *SOCS1 rs243327* was associated with the risk of primary resistance to imatinib in CML. The authors postulated that such functional polymorphism could affect the antagonistic effect of SOCS1 in the *BCR-ABL*-mediated signaling pathways, thus affecting the sensitivity of the leukemic cells to imatinib [92]. Collectively, these observations suggest that SOCS1 levels affect therapeutic response in CML.

In pediatric ALL, downregulation of SOCS3 mRNA expression led to persistent activation of JAK2/STAT3 and concomitant activation of anti-apoptotic response and leukemia progression. Notably, the levels of SOCS3 mRNA correlated with high-risk factors and poor prognosis. Consequently, complete remission patients had upregulation of SOCS3 mRNA and protein expression compared with relapsed and refractory patient samples. In line, *SOCS3* was hypermethylated in ALL samples compared to healthy controls. These observations were correlated with lower SOCS3 transcript and protein levels, and constitutive activation of JAK/STAT3 signaling in the tested ALL samples. SOCS3 hypermethylation was also associated with high levels of Treg cells, which impede intrinsic anti-tumor immunological functions and decrease the clearance of residual tumor cells leading to an increased risk of relapse [71]. These observations position SOCS3 as a potential regulator of residual or relapsed disease in ALL.

Studies have shown that treatment of CLL cells with Hsp90 inhibitor leads to upregulation of SOCS3 mRNA, followed by reduced cell migration to stromal cell-derived factor (SDF-1) and C-X-C motif chemokine (CXCL)13, and increased leukemic cell death [75]. Overexpression of *SOCS3* inhibited CXCL12-induced focal adhesion kinase (FAK) activation and pro-adhesive responses in transduced progenitor B cells. Conversely, *SOCS3*-deficient mice displayed constitutive FAK phosphorylation and adhesion to vascular cell adhesion molecule 1 (VCAM-1) in mature B cells [93]. In line with these reports, *SOCS3* inhibited the CXCL12/CXCR4 signaling axis and reduced the migratory capacity of human AML blasts [79]. Inhibition of CXCR4 was associated with impaired homing and engraftment of AML cells. Importantly, pharmacological blockade of the CXCL12/CXCR4 interaction resulted in leukemic blast mobilization from their protective microenvironment into the peripheral circulation, making them more vulnerable to chemotherapeutic drugs [94].

### 3.2. CIS and SOCS2

*CIS* was identified as an early cytokine responsive gene. In hematopoietic cells, CIS is induced by specific cytokines, including IL-2, IL-3, erythropoietin (EPO), and granulocyte-macrophage colony-stimulating factor (GM-CSF) [95]. CIS serves as a positive regulator of T cell receptor (TCR)-mediated MAPK activation in CD4 + T cells. CIS upregulation promotes TCR-mediated T cell proliferation and cytokine secretion and decreases TCR-driven apoptosis [96]. CIS also negatively regulates IL-3 and EPO-mediated proliferation of M1 myeloid cells [95].

Using integrated genomic and transcriptomic analyses of 1046 childhood B-ALL cases, *CIS* has recently been identified as one of the synergistic key regulators in Philadelphia chromosome-like B-ALL (Ph-like B-ALL) [74]. Deletion of *CIS* in human pluripotent stem cell-derived natural killer cells (*CIS*^-/-^ iPSC-NK) enhanced NK cell cytotoxic activity against K562 and Molm-13 AML cells. In the AML xenograft model, *CIS* depletion in NK cells reduced leukemia burden and increased animal survival. Mechanistically, deletion of *CIS* induced mTOR signaling, leading to an increase in the metabolic fitness of NK cells as seen by enhanced basal glycolysis and glycolytic capacity as well as enhanced maximal mitochondrial respiration, ATP-linked respiration, and spare respiration capacity [80]. Additionally, there is evidence suggesting the role of CIS in negative regulation of Th2 and Treg cell differentiation through disrupting of STAT5 signaling [97,98].

SOCS2, also known as SSI-2 and CIS-2 [99,100], is constitutively expressed in normal peripheral blood mononuclear leukocytes (PBMC). Various cytokines and hormones can induce SOCS2 in several tissues and cell types, including the bone marrow [101,102]. Several reports have identified aberrant expression of SOCS2 in a multitude of cancers, including leukemia [103]. Importantly, *SOCS2* was identified as one of four highly prognostic genes predictive of AML aggressiveness and poor outcome [104]. *SOCS2* mRNA levels were elevated in high-risk AML and ALL harboring *MLL* and *BCR-ABL* chromosomal rearrangements. Upregulation of SOCS2 correlated with the enrichment in hematopoietic and leukemic stemness genes [69,70]. SOCS2 overexpression was also reported in AML cell lines and primary samples carrying *FLT3-ITD* mutations [105]. In addition, higher SOCS2 mRNA levels were associated with poor overall survival in pediatric AML [78]. Functionally, *SOCS2* silencing reduced the growth of *MLL-AF9* transformed murine leukemic cells in vitro and delayed the disease development in a mouse model of *MLL-AF9* driven AML. In contrast, retroviral expression of *SOCS2* increased AML cell proliferation [104].

Radich et al. identified *SOCS2* among the top ten genes associated with CML progression [106]. SOCS2 mRNA was upregulated in CML patients with blast crisis compared with chronic phase patients and healthy individuals. *BCR-ABL* induced *SOCS2* upregulation in CML cell lines, and SOCS2 expression was suppressed in vivo upon *BCR-ABL* tyrosine kinase inhibition [18], suggesting a role of *BCR-ABL* signaling in the regulation of SOCS2 expression. On the contrary, Hansen et al. demonstrated that SOCS2 was dispensable for the development and progression of *BCR-ABL* induced CML. Specifically, the absence of *SOCS2* did not affect the disease latency and the histological presentation of CML in mice transplanted with *BCR-ABL* transduced bone marrow (BM) cells [107]. Collectively, these observations suggest that an insufficient or non-functional *BCR-ABL*-mediated feedback loop leads to the upregulation of *SOCS2* in CML [18,107]. Further studies are required to determine whether and how SOCS2 and potentially other SOCS contribute to CML evolution to blast crisis.

### 3.3. SOCS4 and SOCS5

SOCS4 is the least studied member among the SOCS family members. SOCS4 is constitutively expressed in the thymus of adult pigs and is involved in T cell function and development [108]. Indeed, *SOCS4* mutant mice exhibit defective homing of influenza-specific CD8+ T cells to the lungs and reduced T cell receptor-mediated activation [109]. However, there is no data about the role of SOCS4 in T cell receptor activation in human cells. Accumulating evidence suggests that SOCS4 plays a role in the pathobiology of several cancers [27,28,110,111]. For example, in epithelial cancer cells, the Runx1 transcription factor promotes cancer cell growth via direct repression of *SOCS4* and subsequent activation of STAT3 [112]. To date, the roles of SOCS4 in the regulation of leukemia development and progression remain elusive.

Similar to SOCS4, little is known about the roles of SOCS5 in hematological malignancies. SOCS5 is highly expressed in the spleen, lymph nodes, thymus, and bone marrow, particularly in B and T cells [37,113]. *SOCS5* serves as a tumor suppressor gene in several types of malignancies via its negative regulatory effects on the epidermal growth factor (EGF) receptor and JAK-STAT signaling pathways [114,115]. Our group reported downregulation of SOCS5 mRNA and protein levels in high-risk T-ALL with *KMT2A* rearrangements [72]. In fact, gene expression analyses identified *SOCS5* within the most downregulated genes in high-risk T-ALL samples harboring *KMT2A* rearrangements [116]. Using a T-ALL xenograft model, we demonstrated that *SOCS5* negatively regulates T-ALL progression and extramedullary infiltration. Mechanistically, *SOCS5* depletion induced the activation of JAK-STAT signaling and downregulation of both IL-4 and IL-7 receptors. In addition to T-ALL, downregulation of *SOCS5* mRNA was found in B-ALL and AML primary samples harboring chromosomal alterations involving the *KMT2A* gene [72]. Moreover, forced expression of *KMT2A-MLLT4* and *KMT2A-MLLT1* in Ba/F3 cells reduced SOCS5 protein levels and enhanced JAK-STAT signaling activation [72]. These findings point to the role of SOCS5 in enhancing JAK-STAT signaling in *KMT2A*-rearranged leukemia.

Recent studies have shown that SOCS5 plays a role in CLL-associated immune suppression by impairing dendritic cell (DC) function. Forced expression of *SOCS5* in monocytes isolated from healthy human donors reduced IL-4-mediated STAT6 activation, leading to impaired monocyte differentiation into functionally mature DCs [76]. These studies implicate SOCS5 as a potential therapeutic target for reversing immune suppression in CLL.

### 3.4. SOCS6 and SOCS7

While SOCS6 has been extensively studied over the past few decades, its role in hematological malignancies, including leukemia, has yet to be determined. *SOCS6* functions as a tumor suppressor and its expression is commonly downregulated in a multitude of solid tumors [117,118]. The antitumor effect of SOCS6 relies on its ability to regulate JAK/STAT and PI3K/Akt signaling pathways [31,119]. Studies have shown that upregulation of c-Kit signaling promotes tumor formation and progression of several hematological malignancies, including AML and mast cell leukemia [120,121]. SOCS6 exerted ubiquitin ligase activity on hematopoietic proto-oncogene *c-Kit* and inhibited stem cell factor (SCF)-dependent signaling [122]. Forced expression of *SOCS6* in a Ba/F3-KIT cell line resulted in a significant reduction in SCF-dependent cell proliferation and activation of ERK1/2 and p38 [123].

Furthermore, SOCS6 negatively regulated FMS-related receptor tyrosine kinase 3 (FLT3) and ERK1/2 signaling pathways as well as the cellular proliferation of Ba/F3 and UT-7 cells [124]. In ALL, *SOCS6* mRNA expression was elevated in the BM of newly diagnosed and relapse patient samples compared to patients in complete remission and/or healthy individuals. Another study demonstrated that SOCS6 mRNA levels were positively correlated with chemotherapy-induced remission in ALL patients [73].

Among the SOCS family members, SOCS7 has been given much less attention. SOCS7 is constitutively expressed in many tissues, particularly in the brain and testis [102,125,126]. Its expression in leukocytes is induced by prolactin (PRL), growth hormone (GH), IL-6, and IL-1β [102]. There is evidence for the possible role of SOCS7 in Th2, Th17, and Treg cell development through inhibition of STAT3 and STAT5 signaling [127].

Limited data is available regarding the roles of SOCS7 in cancer. Tumor suppressive effects of SOCS7 were reported in the prostate [128], colon [129], and breast cancer [19]. SOCS7 regulated the activation of the tumor suppressor P53 and induced cell cycle arrest in response to DNA damage [41]. Elevated SOCS7 mRNA levels were inversely correlated with TNM and tumor stages of breast cancer consistent with better disease-free survival and overall survival [19]. However, there are no reports regarding the biological role of SOCS7 in leukemia.

## 4. Regulation of SOCS Expression and Functions

### 4.1. Epigenetic Dysregulation of SOCS Genes

Aberrant DNA methylation of promoter CpG islands of tumor suppressor genes is a prevalent cancer phenomenon. It leads to tumor suppressor gene silencing and, as a result, a downregulation that significantly contributes to tumorigenesis [130]. Epigenetic dysregulation of *SOCS* has been identified in various malignancies [71,130,131]. Aberrant methylation resulting in *SOCS1* downregulation was reported in hepatocellular carcinoma [131]. *SOCS1* hypermethylation enhanced IL-6-mediated cell proliferation in pancreatic adenocarcinoma [132] and was positively correlated with lymph node metastasis and a low survival rate in colorectal cancer [133]. *SOCS3* hypermethylation was linked to constitutive activation of STAT3 and unfavorable clinical outcomes in prostate cancer [25] and to enhanced epithelial cell proliferation in gastric cancer [134]. Similarly, epigenetic silencing of *SOCS6* through promoter hypermethylation increased gastric cancer cell proliferation and inhibited apoptotic cell death [32]. Hypermethylation of *SOCS2* was reported in 14% of primary ovarian cancers [130], 25% of colorectal cancer [135], and 80% of tested melanoma cell lines [136]. Downregulation of *SOCS4* expression corresponding to CpG islands hypermethylation of the *SOCS4* was also found in gastric cancer [110].

In the context of leukemia, *SOCS1* methylation has been documented in a variety of leukemia subtypes, including about 60% of newly diagnosed AML [16]. *SOCS1* hypermethylation and its increased ubiquitin-mediated degradation were proposed as the principal mechanism underlying SOCS1 downregulation in AML primary samples and cell lines [88,137,138]. Furthermore, *SOCS1* methylation induced IL-3 expression in leukemic cells promoting their resistance to imatinib and cytotoxic T cells in the BCR-ABL DA1-3b mouse model of AML [139]. Aberrant methylation of *SOCS1* was also associated with constitutive activation of JAK/STAT signaling, increased leukemic stem cell proliferation, and poor prognosis in CML [81,82]. CML patients in blast crisis and chronic phase had *SOCS1* methylation that was reversed to unmethylated status during the remission phase [91]. Constitutive activation of STAT3 due to *SOCS1* and *SOCS3* hypermethylation increased proliferation of *BCR-ABL* positive CML cell lines resistant to imatinib [81]. Pena et al. reported *SOCS1* exon-2 methylation as a frequent event in primary CML samples (46.6%) compared to *SOCS1* promoter region methylation (6.6%) [140]. Contrasting results were reported by Hatirnaz et al. who identified hypomethylation of *SOCS1* gene exon-2 in CML patients [90]. In pediatric ALL samples, *SOCS3* methylation led to constitutive activation of JAK/STAT3 signaling and enhanced Treg cell expression, which in turn negatively regulated anti-tumor immunity. Hypomethylation of *SOCS3* was reported in complete remission in ALL patients compared with increased *SOCS3* methylation found in relapsed and refractory ALL samples [71]. *SOCS3* hypermethylation was also linked to imatinib resistance in *BCR-ABL* positive CML cell lines [81]. However, Elias et al. excluded *SOCS1* hypermethylation as a predictor of CML resistance to tyrosine kinase inhibitors [141]. Beyond that, *SOCS5* hypermethylation and resulting SOCS5 mRNA and protein downregulation potentiated proliferation of T-ALL cell lines and disease progression in T-ALL xenograft models. Furthermore, in T-ALL cell lines and primary samples, *SOCS5* expression was regulated by histone deacetylation through the recruitment of the methyl CpG binding protein 2 (MeCP2) and SIN3 co-repressor complex [72].

In addition to epigenetic regulation, polymorphism in *SOCS1* and *SOCS2* was shown in ALL primary samples. The *SOCS2* *rs3816997 AC* genotype was associated with decreased SOCS2 mRNA expression and high susceptibility to ALL compared with the CC genotype. Conversely, the *SOCS1* *rs33977706 CA* genotype was linked to lower susceptibility to ALL [89]. In another example, the *SOCS1* *rs243327* SNP correlated with the risk of primary resistance to imatinib in newly diagnosed CML [92].

### 4.2. MicroRNA Regulation of SOCS Genes

MicroRNAs (miRNAs) are small (~22 nucleotides) noncoding RNA that bind with 3’ untranslated regions (UTR) on target mRNA leading to transitional repression [142]. Bioinformatics studies have indicated that each miRNA can regulate hundreds of target genes thus affecting critical cellular processes such as proliferation, migration, differentiation, and apoptosis [143]. Emerging evidence suggests the role of miRNAs in the regulation of hematopoiesis, leukemia development, and progression [144]. However, relatively little is known about the regulatory role of miRNAs on SOCS expression in leukemia. In this section, we summarize the current knowledge regarding the effects of miRNAs on SOCS expression and their impact on the development and progression of leukemia.

MiR-155 functions as an oncomiR in various types of cancer, including hematological malignancies [145,146,147,148]. Indeed, *SOCS1* was identified as a potential target of miR-155 [149]. In hematopoietic cells, miR-155 modulated Treg and Th17 cell differentiation and IL-17A production by targeting SOCS1 [150]. Overexpression of miR-155 resulted in constitutive activation of JAK/STAT3 signaling and increased cell proliferation of breast cancer cells by targeting *SOCS1* [20]. Interestingly, miR-155 was also upregulated in AML primary samples and in a mouse model of lymphoma, suggesting its potential role in hematological malignancies [151,152]. Murine double minute 2 (MDM2) small molecule inhibitor Nutlin-3 significantly enhanced *SOCS1* mRNA levels concurrent with downregulation of miR-155 levels in both, primary B-CLL cells and B-CLL cell lines in vitro [153]. In addition, inhibition of miR-19 and miR-155 reactivated the expression of SOCS1 and p53 in mouse leukemia and human myeloma cells, leading to subsequent inhibition of cell proliferation, cell migration, and tumor progression [154]. Furthermore, miR-311 was upregulated in primary ALL and CLL samples compared with healthy individuals, and *SOCS1* was identified as one of its putative targets [155]. In other studies, miR-19a induced proliferation and tumorigenesis of gastric cancer and non-small cell lung cancer cells by targeting *SOCS1* [156,157]. Moreover, miR19a/b downregulated *SOCS1* expression in multiple myeloma cell lines in vitro. Silencing of miR19a/b expression resulted in significant tumor suppression in mice xenografted with multiple myeloma cell lines [158]. Functionally, miR-19a also downregulated SOCS3 mRNA and protein expression and enhanced IFN-α and IL-6 signaling in vitro [159]. MiR-221 directly inhibited *SOCS3* expression and enhanced IFN-γ sensitivity in prostate cancer cells [160]. Following the same trend, miR-30a-5p promoted cholangiocarcinoma progression by targeting SOCS3-mediated signaling [161]. Upregulation of *SOCS3* led to enhanced miR-124-3p expression in CML cell lines [162]. MiR-183-5p has been identified as a post-transcriptional regulator of *SOCS6*. Silencing of miR-183-5p increased *SOCS6* expression and decreased pancreatic cancer cell growth and motility in vitro [163]. Upregulation of miR-486 was found in primary AML samples. Consequently, miR-486 accelerated STAT3 nuclear translocation and JAK-STAT signaling by direct downregulation of *SOCS2* expression in AML cell lines in vitro [164]. A recent investigation showed that repression of *SOCS5* by miR-18a-5p decreased cell proliferation and migration, and induced apoptosis in CML cell lines and in the xenograft model of CML [165]. In addition, the growth-suppressive effect of miR-101 was identified in human *Helicobacter pylori*-related gastric cancer through repression of *SOCS2* [166].

## 5. Concluding Remarks

The dysregulation of SOCS has been implicated in various tumor types through different mechanisms. Because SOCS are known negative regulators of cytokine signaling, their downregulation could significantly impact the cytokine-driven cell signaling pathways leading to constitutive activation of JAK/STAT and other pathways, thus promoting oncogenic transformation, tumor invasion, and metastasis. Dysregulation of SOCS is also seen in various leukemias (Table 2). While SOCS have been emerging as functional tumor suppressors in leukemia pathobiology and their silencing has been associated with unfavorable prognosis, their function in leukemogenesis seems complex and likely leukemia-type dependent.

These contradictory data on the roles of SOCS in cancer and leukemia could be attributed to the differences in the tumor origin, tumor microenvironment, and genetic makeup of the cancer cells. Thus, further genetic and functional studies are warranted to explore the mechanistic roles of various SOCS proteins in leukemogenesis. These studies will develop new insights into the roles played by negative regulators of cytokine signaling in the pathobiology of leukemia and will lead to the discovery of novel prognostic or therapeutic targets.

## Figures and Tables

**Table 1:** Contrasting roles of suppressors of cytokine signaling (SOCS) in the development of cancer.

Gene	Cancer Type	Expression	Function	Ref.
*SOCS1*	Breast cancer	Up-regulation	Associated with better clinical outcomes	[19]
		Down-regulation	Enhances cell proliferation and colony formation	[20]
	Colorectal tumor	Up-regulation	Reduces tumor cell invasion	[21]
	Multiple myeloma	Down-regulation	Supports the survival and expansion of multiple myeloma cells	[22]
	Prostate cancer	Down-regulation	Associated with regional lymph node invasion	[23]
*SOCS2*	Hepatocellular carcinoma	Down-regulation	Associated with aggressive tumor progression and poor prognosis	[15]
	Prostate cancer	Down-regulation	Promotes cancer metastasis	[24]
*SOCS3*	Prostate cancer	Down-regulation	Associated with unfavorable clinical outcome	[25]
	Colorectal cancer	Up-regulation	Inhibits proliferation, migration, and invasion, while increasing cell apoptosis	[26]
*SOCS4*	Thyroid cancer	Down-regulation	Induces cell migration and invasion	[27]
	Renal cancer	Down-regulation	Promotes cells proliferation and migration	[28]
*SOCS5*	Pancreatic cancer	Down-regulation	Promotes tumor growth, invasion, and metastasis	[29]
	Hepatocellular carcinoma	Down-regulation	Induces autophagy, reduces cell invasion and metastasis	[30]
*SOCS6*	Breast cancer	Down-regulation	Promotes cell proliferation, tumor growth and induces tamoxifen resistance	[31]
	Gastric cancer	Down-regulation	Inhibits cell proliferation and colony formation	[32]
	Hepatocellular carcinoma	Down-regulation	Induces aggressive tumor progression and poor prognosis	[15]
*SOCS7*	Bladder cancer	Up-regulation	Induces tumor growth	[33]
	Breast cancer	Down-regulation	Increases tumor growth and migration	[34]
*CIS*	Breast cancer	Up-regulation	Increases cell proliferation	[17]

**Table 2:** Biological and clinical significance of the suppressors of cytokine signaling (SOCS) in leukemia.

Leukemia type	Gene	Expression	Function	Ref
**Lymphocytic leukemia**				
**ALL**	*SOCS2*	Up-regulation	Correlated with the enrichment in hematopoietic and leukemic stemness genes.	[69,70]
	*SOCS3*	Down-regulation	Associated with constitutive activation of JAK/STAT3 signaling and negatively regulated anti-tumor immunity.	[71]
	*SOCS5*	Down-regulation	Associated with T-ALL and B-ALL harboring *KMT2A* rearrangements.	[72]
	*SOCS6*	Up-regulation	Negatively correlated with chemotherapy-induced remission in ALL patients.	[73]
	*CIS*		Identified as one of the synergistic key regulators in Ph-like B-ALL.	[74]
**CLL**	*SOCS3*	Down-regulation	Forced expression of SOCS3 reduced cell migration and increased leukemic cell death.	[75]
	*SOCS5*	Up-regulation	Associated with immune suppression in CLL.	[76]
**Myelogenous leukemia**				
**AML**	*SOCS1*	Down-regulation	Associated with relapsed/refractory AML compared to remission and normal control samples.	[77]
	*SOCS2*	Up-regulation	Associated with poor overall survival in pediatric AML.	[78]
	*SOCS3*		Inhibited the CXCL12/CXCR4 signaling axis and reduced the migratory capacity of AML blasts.	[79]
	*SOCS5*	Down-regulation	Associated with AML samples harboring KMT2A rearrangements.	[72]
	*CIS*		Deletion of *CIS* in human pluripotent stem cell-derived natural killer cells enhanced anti-tumor immunity	[80]
**CML**	*SOCS1*	Down-regulation	Associated with constitutive activation of JAK/STAT signaling, increased leukemic stem cell proliferation, and poor prognosis.	[81,82]
		UP-regulation	Subverted cytogenetic response to IFN-α and linked to poor prognosis.	[83]
	*SOCS2*	Up-regulation	Associated with blast crisis compared with chronic phase patients and healthy individuals	[18]
	*SOCS3*	Down-regulation	Linked to imatinib resistance in BCR-ABL positive CML	[81]

## Data Availability

Not applicable.

## References

[1] Van Etten R.A. (2007). Aberrant Cytokine Signaling in Leukemia. Oncogene.

[2] Shi Y., Zhang Z., Qu X., Zhu X., Zhao L., Wei R., Guo Q., Sun L., Yin X., Zhang Y. (2018). Roles of STAT3 in Leukemia (Review). Int. J. Oncol..

[3] Chaudhari S., Desai J.S., Adam A., Mishra P.R. (2014). JAK/STAT as a Novel Target for Treatment of Leukemia. Int. J. Pharm. Pharm. Sci..

[4] Mirantes C., Passegue E., Pietras E.M. (2014). Pro-inflammatory cytokines: Emerging Players Regulating HSC Function in Normal and Diseased Hematopoiesis. Exp. Cell Res..

[5] Murray P.J. (2007). The JAK-STAT Signaling Pathway: Input and Output Integration. J. Immunol..

[6] Malemud C., Pearlman E. (2009). Targeting JAK/STAT Signaling Pathway in Inflammatory Diseases. Curr. Signal. Transduct. Ther..

[7] Ram P.T., Iyengar R. (2001). G Protein Coupled Receptor Signaling through the Src and Stat3 Pathway: Role in Proliferation and Transformation. Oncogene.

[8] Bowman T., Garcia R., Turkson J., Jove R. (2000). STATs in Oncogenesis. Oncogene.

[9] Jiang M., Zhang W.W., Liu P., Yu W., Liu T., Yu J. (2017). Dysregulation of SOCS-Mediated Negative Feedback of Cytokine Sig-naling in Carcinogenesis and Its Significance in Cancer Treatment. Front. Immunol..

[10] Wormald S., Hilton D. (2004). Inhibitors of Cytokine Signal Transduction. J. Biol. Chem..

[11] A Weniger M., Melzner I., Menz C.K., Wegener S., Bucur A.J., Dorsch K., Mattfeldt T., E Barth T.F., Möller P. (2006). Mutations of the Tumor Suppressor Gene SOCS-1 in Classical Hodgkin Lymphoma are Frequent and Associated with Nuclear Phospho-STAT5 Accumulation. Oncogene.

[12] Mottok A., Renné C., Seifert M., Oppermann E., Bechstein W., Hansmann M.-L., Küppers R., Bräuninger A. (2009). Inactivating SOCS1 Mutations are Caused by Aberrant Somatic Hypermutation and Restricted to a Subset of B-Cell Lymphoma Entities. Blood.

[13] Melzner I., Bucur A.J., Brüderlein S., Dorsch K., Hasel C., Barth T.F.E., Leithäuser F., Möller P. (2005). Biallelic Mutation of SOCS-1 Impairs JAK2 Degradation and Sustains Phospho-JAK2 Action in the MedB-1 Mediastinal Lymphoma Line. Blood.

[14] Yang M., Chen H., Zhou L., Huang X., Su F., Wang P. (2020). Identification of SOCS Family Members with Prognostic Values in Human Ovarian Cancer. Am. J. Transl. Res..

[15] Qiu X., Zheng J., Guo X., Gao X., Liu H., Tu Y., Zhang Y. (2013). Reduced Expression of SOCS2 and SOCS6 in Hepatocellular Carcinoma Correlates with Aggressive Tumor Progression and Poor Prognosis. Mol. Cell. Biochem..

[16] Chen C.-Y., Tsay W., Tang J.-L., Shen H.-L., Lin S.-W., Huang S.-Y., Yao M., Chen Y.-C., Shen M.-C., Wang C.-H. (2003). SOCS1 Methylation in Patients with Newly Diagnosed Acute Myeloid Leukemia. Genes Chromosom. Cancer.

[17] Raccurt M., Tam S.P., Lau P., Mertani H.C., Lambert A., Garcia-Caballero T., Li H., Brown R.J., A McGuckin M., Morel G. (2003). Suppressor of Cytokine Signalling Gene Expression is Elevated in Breast Carcinoma. Br. J. Cancer.

[18] Schultheis B., Carapeti-Marootian M., Hochhaus A., Weiβer A., Goldman J.M., Melo J.V. (2002). Overexpression of SOCS-2 in Advanced Stages of Chronic Myeloid Leukemia: Possible Inadequacy of a Negative Feedback Mechanism. Blood.

[19] Sasi W., Jiang W.G., Sharma A., Mokbel K. (2010). Higher Expression Levels of SOCS 1,3,4,7 are Associated with Earlier Tumour Stage and better Clinical Outcome in Human Breast Cancer. BMC Cancer.

[20] Jiang S., Zhang H.-W., Lu M.-H., He X.-H., Li Y., Gu H., Liu M.-F., Wang E.-D. (2010). MicroRNA-155 Functions as an OncomiR in Breast Cancer by Targeting the Suppressor of Cytokine Signaling 1 Gene. Cancer Res..

[21] David M., Naudin C., Letourneur M., Polrot M., Renoir J.-M., Lazar V., Dessen P., Roche S., Bertoglio J., Pierre J. (2014). Suppressor of Cytokine Signaling 1 Modulates Invasion and Metastatic Potential of Colorectal Cancer Cells. Mol. Oncol..

[22] Galm O., Yoshikawa H., Esteller M., Osieka R., Herman J.G. (2003). SOCS-1, A Negative Regulator of Cytokine Signaling, is Fre-quently Silenced by Methylation in Multiple Myeloma. Blood.

[23] Chevrier M., Bobbala D., Villalobos-Hernandez A., Khan G.M., Ramanathan S., Saucier C., Ferbeyre G., Geha S., Ilangumaran S. (2017). Expression of SOCS1 and the Downstream Targets of Its Putative Tumor Suppressor Functions in Prostate Cancer. BMC Cancer.

[24] Das R., Gregory P., Fernandes R.C., Denis I., Wang Q., Townley S.L., Zhao S.G., Hanson A.R., Pickering M.A., Armstrong H. (2016). MicroRNA-194 Promotes Prostate Cancer Metastasis by Inhibiting SOCS2. Cancer Res..

[25] Pierconti F., Martini M., Pinto F., Cenci T., Capodimonti S., Calarco A., Bassi P.F., Larocca L.M. (2010). Epigenetic Silencing of SOCS3 Identifies a Subset of Prostate Cancer with an Aggressive Behavior. Prostate.

[26] Chu Q., Shen D., He L., Wang H., Liu C., Zhang W. (2017). Prognostic Significance of SOCS3 and Its Biological Function in Col-orectal Cancer. Gene.

[27] Mei Z., Chen S., Chen C., Xiao B., Li F., Wang Y., Tao Z. (2015). Interleukin-23 Facilitates Thyroid Cancer Cell Migration and Invasion by Inhibiting SOCS4 Expression via MicroRNA-25. PLoS ONE.

[28] Song W.B., Chen Y.L., Zhu G.D., Xie H.J., Yang Z.S., Li L. (2020). Exosome-Mediated miR-9-5p Promotes Proliferation and Mi-gration of Renal Cancer Cells both In Vitro and In Vivo by Targeting SOCS4. Biochem. Biophys. Res. Commun..

[29] Hu H., Zhang Q., Chen W., Wu T., Liu S., Li X., Luo B., Zhang T., Yan G., Lu H. (2019). MicroRNA-301a Promotes Pancreatic Cancer Invasion and Metastasis through the JAK/STAT3 Signaling Pathway by Targeting SOCS5. Carcinogenesis.

[30] Zhang M., Liu S., Chua M.-S., Li H., Luo D., Wang S., Zhang S., Han B., Sun C. (2019). SOCS5 Inhibition Induces Autophagy to Impair Metastasis in Hepatocellular Carcinoma Cells via the PI3K/Akt/mTOR Pathway. Cell Death Dis..

[31] Shen R., Wang Y., Wang C.-X., Yin M., Liu H.-L., Chen J.-P., Han J.-Q., Wang W.-B. (2015). MiRNA-155 Mediates TAM Resistance by Modulating SOCS6-STAT3 Signalling Pathway in Breast Cancer. Am. J. Transl. Res..

[32] Lai R.-H., Hsiao Y.-W., Wang M.-J., Lin H.-Y., Wu C.-W., Chi C.-W., Li A.F.-Y., Jou Y.-S., Chen J.-Y. (2010). SOCS6, Down-Regulated in Gastric Cancer, Inhibits Cell Proliferation and Colony Formation. Cancer Lett..

[33] Noguchi S., Yamada N., Kumazaki M., Yasui Y., Iwasaki J., Naito S. (2013). socs7, A Target Gene of microRNA-145, Reg-ulates Interferon-β Induction Through STAT3 Nuclear Translocation in Bladder. Cancer Cells.

[34] Sasi W., Ye L., Jiang W.G., Mokbel K., Sharma A. (2013). Observations on The effects of Suppressor of Cytokine Signaling 7 (SOCS7) Knockdown in Breast Cancer Cells: Their In Vitro response to Insulin Like Growth Factor I (IGF-I). Clin. Transl. Oncol..

[35] Huang S., Liu K., Cheng A., Wang M., Cui M., Huang J., Zhu D., Chen S., Liu M., Zhao X. (2020). SOCS Proteins Participate in the Regulation of Innate Immune Response Caused by Viruses. Front. Immunol..

[36] Yoshimura A., Naka T., Kubo M. (2007). SOCS Proteins, Cytokine Signalling and Immune Regulation. Nat. Rev. Immunol..

[37] Trengove M.C., Ward A.C. (2013). SOCS Proteins in Development and Disease. Am. J. Clin. Exp. Immunol..

[38] Ahmed C.M.I., Larkin J., Johnson H.M. (2015). SOCS1 Mimetics and Antagonists: A Complementary Approach to Positive and Negative Regulation of Immune Function. Front. Immunol..

[39] Feng Z.-P., Chandrashekaran I., Low A., Speed T.P., Nicholson S.E., Norton R.S. (2011). The N-Terminal Domains of SOCS Proteins: A Conserved Region in the Disordered N-Termini of SOCS4 and 5. Proteins: Struct. Funct. Bioinform..

[40] Hwang M.N., Min C.H., Kim H.S., Lee H., Yoon K.A., Park S.Y., Lee E.S., Yoon S. (2007). The Nuclear Localization of SOCS6 Requires the N-Terminal Region and Negatively Regulates Stat3 Protein Levels. Biochem Biophys Res. Commun..

[41] Kremer B.E., Adang L.A., Macara I.G. (2007). Septins Regulate Actin Organization and Cell-Cycle Arrest through Nuclear Accumulation of NCK Mediated by SOCS7. Cell.

[42] Kubo M., Hanada T., Yoshimura A. (2003). Suppressors of Cytokine Signaling and Immunity. Nat. Immunol..

[43] Bullock A.N., Rodriguez M.C., Debreczeni J., Songyang Z., Knapp S. (2007). Structure of the SOCS4-ElonginB/C Complex Reveals a Distinct SOCS Box Interface and the Molecular Basis for SOCS-Dependent EGFR Degradation. Structure.

[44] Linossi E.M., Calleja D.J., Nicholson S.E. (2018). Understanding SOCS Protein Specificity. Growth Factors.

[45] Krebs D.L., Hilton D. (2001). SOCS Proteins: Negative Regulators of Cytokine Signaling. STEM Cells.

[46] Zhang J.-G., Farley A., Nicholson S.E., Willson T.A., Zugaro L.M., Simpson R., Moritz R.L., Cary D., Richardson R., Hausmann G. (1999). The Conserved SOCS box Motif in Suppressors of Cytokine Signaling Binds to Elongins B and C and May Couple bound Proteins to Proteasomal Degradation. Proc. Natl. Acad. Sci. USA.

[47] Kamura T., Maenaka K., Kotoshiba S., Matsumoto M., Kohda D., Conaway R.C., Conaway J., Nakayama K.I. (2004). VHL-box and SOCS-box Domains Determine Binding Specificity for Cul2-Rbx1 and Cul5-Rbx2 Modules of Ubiquitin Ligases. Genes Dev..

[48] Kohroki J., Nishiyama T., Nakamura T., Masuho Y. (2005). ASB Proteins Interact with Cullin5 and Rbx2 to form E3 Ubiquitin Ligase Complexes. FEBS Lett..

[49] Gianfelici V., Chiaretti S., Demeyer S., Di Giacomo F., Messina M., La Starza R., Peragine N., Paoloni F., Geerdens E., Pierini V. (2016). RNA Sequencing Unravels the Genetics of Refractory/Relapsed T-cell Acute Lymphoblastic Leukemia. Prognostic and Therapeutic Implications. Haematology.

[50] Lin T.S., Mahajan S., A Frank D. (2000). STAT Signaling in The Pathogenesis and Treatment of Leukemias. Oncogene.

[51] Girardi T., Vereecke S., O Sulima S., Khan Y., Fancello L., Briggs J.W., Schwab C., De Beeck J.O., Verbeeck J., Royaert J. (2017). The T-cell Leukemia-Associated Ribosomal RPL10 R98S Mutation Enhances JAK-STAT Signaling. Leukemia.

[52] Gouilleux-Gruart V., Gouilleux F., Desaint C., Claisse J.F., Capiod J.C., Delobel J. (1996). STAT-Related Transcription Factors are Con-stitutively Activated in Peripheral Blood Cells from Acute Leukemia Patients. Blood.

[53] Ikezoe T., Kojima S., Furihata M., Yang J., Nishioka C., Takeuchi A., Isaka M., Koeffler H.P., Yokoyama A. (2011). Expression of p-JAK2 Predicts Clinical Outcome and is a Potential Molecular Target of Acute Myelogenous Leukemia. Int. J. Cancer.

[54] Benekli M., Xia Z., Donohue K.A., Ford L.A., Pixley L.A., Baer M.R., Baumann H., Wetzler M. (2002). Constitutive Activity of Signal Transducer and Activator of Transcription 3 Protein in Acute Myeloid Leukemia Blasts is Associated with Short Disease-Free Survival. Blood.

[55] Chakraborty A., White S.M., Schaefer T.S., Ball E.D., Dyer K.F., Tweardy D.J. (1996). Granulocyte Colony-Stimulating Factor Activation of Stat3 Alpha and Stat3 Beta in Immature Normal and Leukemic Human Myeloid Cells. Blood.

[56] Cook A., Li L., Ho Y., Lin A., Stein A., Forman S., Perrotti D., Jove R., Bhatia R. (2014). Role of Altered Growth Factor Receptor-Mediated JAK2 Signaling in Growth and Maintenance of Human Acute Myeloid Leukemia Stem Cells. Blood.

[57] De Bock C.E., Demeyer S., Degryse S., Verbeke D., Sweron B., Gielen O., Vandepoel R., Vicente C., Bempt M.V., Dagklis A. (2018). HOXA9 Cooperates with Activated JAK/STAT Signaling to Drive Leukemia Development. Cancer Discov..

[58] Roncero A.M., Nieva P.L., Cobos-Fernández M.A., Villa-Morales M.C., Gonzalez-Sanchez L., López-Lorenzo J.L., Llamas P., Ayuso C., Rodriguezpinilla S.M., Arriba M.C. (2016). Contribution of JAK2 Mutations to T-Cell Lymphoblastic Lymphoma Development. Leukemia.

[59] Flex E., Petrangeli V., Stella L., Chiaretti S., Hornakova T., Knoops L., Ariola C., Fodale V., Clappier E., Paoloni F. (2008). Somatically Acquired JAK1 Mutations in Adult Acute Lymphoblastic Leukemia. J. Exp. Med..

[60] Tomasson M.H., Xiang Z., Walgren R., Zhao Y., Kasai Y., Miner T., Ries R., Lubman O., Fremont D.H., McLellan M.D. (2008). Somatic Mutations and Germline Sequence Variants in The Expressed Tyrosine Kinase Genes of Patients with de Novo Acute Myeloid Leukemia. Blood.

[61] Degryse S., Bornschein S., De Bock C.E., Leroy E., Bempt M.V., Demeyer S., Jacobs K., Geerdens E., Gielen O., Soulier J. (2018). Mutant JAK3 Signaling is Increased by Loss of Wild-Type JAK3 or by Acquisition of Secondary JAK3 Mutations in T-ALL. Blood.

[62] Zenatti P.P., Ribeiro D., Li W., Zuurbier L., da Silva M.C., Paganin M., Tritapoe J., A Hixon J., Silveira A., Cardoso B. (2011). Oncogenic IL7R Gain-of-Function Mutations in Childhood T-Cell Acute Lymphoblastic Leukemia. Nat. Genet..

[63] Mangolini M., De Boer J., Walf-Vorderwülbecke V., Pieters R., Boer M.L.D., Williams O. (2013). STAT3 Mediates Oncogenic Addiction to TEL-AML1 in t(12;21) Acute Lymphoblastic Leukemia. Blood.

[64] Gianfelici V., Messina M., Paoloni F., Peragine N., Lauretti A., Fedullo A.L., Di Giacomo F., Vignetti M., Vitale A., Guarini A. (2018). IL7R Overexpression in Adult Acute Lymphoblastic Leukemia is Associated to JAK/STAT Pathway Mutations and Identifies Patients who Could Benefit from Targeted Therapies. Leuk. Lymphoma.

[65] Govaerts I., Jacobs K., Vandepoel R., Cools J. (2019). JAK/STAT Pathway Mutations in T-ALL, Including the STAT5B N642H Mutation, are Sensitive to JAK1/JAK3 Inhibitors. HemaSphere.

[66] Maude S.L., Dolai S., Delgado-Martin C., Vincent T., Robbins A., Selvanathan A., Ryan T., Hall J., Wood A.C., Tasian S.K. (2015). Efficacy of JAK/STAT Pathway Inhibition in Murine Xenograft Models of Early T-Cell Precursor (ETP) Acute Lymphoblastic Leukemia. Blood.

[67] Zhang M., Griner L.A.M., Ju W., Duveau D.Y., Guha R., Petrus M.N. (2015). Selective Targeting of JAK/STAT Signaling is Potentiated by Bcl-xL Blockade in IL-2–Dependent Adult T-Cell Leukemia. Proc. Natl. Acad. Sci. USA.

[68] Vainchenker W., Constantinescu S. (2012). JAK/STAT Signaling in Hematological Malignancies. Oncogene.

[69] Vitali C., Bassani C., Chiodoni C., Fellini E., Guarnotta C., Miotti S. (2015). SOCS2 Controls Proliferation and Stemness of Hema-topoietic Cells under Stress Conditions and Its Deregulation Marks Unfavorable Acute Leukemias. Cancer Res..

[70] Abdelrasoul H., Vadakumchery A., Werner M., Lenk L., Khadour A., Young M., El Ayoubi O., Vogiatzi F., Krämer M., Schmid V. (2020). Synergism between IL7R and CXCR4 Drives BCR-ABL Induced Transformation in Philadelphia Chromosome-Positive Acute Lymphoblastic Leukemia. Nat. Commun..

[71] Liu K., Wu Z., Chu J., Yang L., Wang N. (2019). Promoter Methylation and Expression of SOCS3 Affect The Clinical Outcome of Pediatric Acute Lymphoblastic Leukemia by JAK/STAT Pathway. Biomed. Pharmacother..

[72] Sharma N.D., Nickl C.K., Kang H., Ornatowski W., Brown R., Ness S., Loh M.L., Mullighan C.G., Winter S.S., Hunger S.P. (2019). Epigenetic Silencing of SOCS 5 Potentiates JAK-STAT Signaling and Progression of T-Cell Acute Lymphoblastic Leukemia. Cancer Sci..

[73] Liu J., Zheng Y., Gao J., Zhu G., Gao K., Zhang W., Shi F., Zhang Q. (2017). Expression of SHP-1 and SOCS6 in Patients with Acute Leukemia and their Clinical Implication. OncoTargets Ther..

[74] Ding Y.-Y., Kim H., Madden K., Loftus J.P., Chen G.M., Allen D.H., Zhang R., Xu J., Chen C.-H., Hu Y. (2021). Network Analysis Reveals Synergistic Genetic Dependencies for Rational Combination Therapy in Philadelphia Chromosome-like Acute Lymphoblastic Leukemia. Clin. Cancer Res..

[75] Chen T.L., Gupta N., Lehman A., Ruppert A.S., Yu L., Oakes C.C., Claus R., Plass C., Maddocks K.J., Andritsos L. (2016). Hsp90 Inhibition Increases SOCS3 Transcript and Regulates Migration and Cell Death in Chronic Lymphocytic Leukemia. Oncotarget.

[76] Toniolo P.A., Liu S., Yeh J.E., Ye D.Q., Barbuto J.A.M., Frank D.A. (2016). Deregulation of SOCS5 Suppresses Dendritic Cell Function in Chronic Lymphocytic Leukemia. Oncotarget.

[77] Zhang X., Yang L., Liu X., Zhan Y., Pan Y., Wang X., Luo J. (2018). Association between Methylation of Tumor Suppressor Gene SOCS1 and Acute Myeloid Leukemia. Oncol. Rep..

[78] Laszlo G.S., Ries R.E., Gudgeon C.J., Harrington K.H., Alonzo T.A., Gerbing R.B. (2014). High Expression of Suppressor of Cytokine Signaling-2 Predicts Poor Outcome in Pediatric Acute Myeloid Leukemia: A Report from the Children’s Oncology Group. Leuk. Lymphoma.

[79] Jacobia A., Thiemea S., Lehmann R., Ugarte F., Malech H., Koch S., Thiede C., Müller K., Bornhäuser M., Ryser M. (2010). Impact of CXCR4 Inhibition on FLT3-ITD−Positive Human AML Blasts. Exp. Hematol..

[80] Zhu H., Blum R.H., Bernareggi D., Ask E.H., Wu Z., Hoel H.J. (2020). Metabolic Reprograming via Deletion of CISH in Human iPSC-Derived NK Cells Promotes In Vivo Persistence and Enhances Anti-tumor Activity. Cell Stem Cell..

[81] Al-Jamal H., Jusoh S.A.M., Yong A.C., Asan J.M., Hassan R., Johan M.F. (2014). Silencing of Suppressor of Cytokine Signaling-3 due to Methylation Results in Phosphorylation of STAT3 in Imatinib Resistant BCR-ABL Positive Chronic Myeloid Leukemia Cells. Asian Pac. J. Cancer Prev..

[82] Behzad M.M., Shahrabi S., Jaseb K., Bertacchini J., Ketabchi N., Saki N. (2018). Aberrant DNA Methylation in Chronic Myeloid Leukemia: Cell Fate Control, Prognosis, and Therapeutic Response. Biochem. Genet..

[83] Roman-Gomez J., Jimenez-Velasco A., Castillejo J.A., Cervantes F., Barrios M., Colomer D. (2004). The Suppressor of Cytokine Signaling-1 is Constitutively Expressed in Chronic Myeloid Leukemia and Correlates with Poor Cytogenetic Response to Interfer-on-Alpha. Haematologica.

[84] Zhu Z., Lu X., Jiang L., Sun X., Zhou H., Jia Z., Zhang X., Ma L. (2015). STAT3 Signaling Pathway is Involved in Decitabine Induced Biological Phenotype Regulation of Acute Myeloid Leukemia Cells. Am. J. Transl. Res..

[85] Teramo A., Gattazzo C., Passeri F., Lico A., Tasca G., Cabrelle A., Martini V., Frezzato F., Trimarco V., Ave E. (2013). Intrinsic and Extrinsic Mechanisms Contribute to Maintain the JAK/STAT Pathway Aberrantly Activated in T-Type Large Granular Lymphocyte Leukemia. Blood.

[86] Rottapel R., Ilangumaran S., Neale C., La Rose J., Ho J.M.-Y., Nguyen M.H.-H., Barber D., Dubreuil P., De Sepulveda P. (2002). The Tumor Suppressor Activity of SOCS-1. Oncogene.

[87] Frantsve J., Schwaller J., Sternberg D.W., Kutok J., Gilliland D.G. (2001). Socs-1 Inhibits TEL-JAK2-Mediated Transformation of Hemato-Poietic Cells through Inhibition of JAK2 Kinase Activity and Induction of Proteasome-Mediated Degradation. Mol. Cell. Biol..

[88] Watanabe D., Ezoe S., Fujimoto M., Kimura A., Saito Y., Nagai H., Tachibana I., Matsumura I., Tanaka T., Kanegane H. (2004). Suppressor of Cytokine Signalling-1 Gene Silencing in Acute Myeloid Leukaemia and Human Haematopoietic Cell Lines. Br. J. Haematol..

[89] Chen S.-S., Wu W.-Z., Zhang Y.-P., Huang W.-J. (2020). Gene Polymorphisms of SOCS1 and SOCS2 and Acute Lymphoblastic Leukemia. Eur. Rev. Med. Pharmacol. Sci..

[90] Ng O.H., Ure U., Ar M.C., Akyerli C., Soysal T., Ferhanoğlu B., Özçelik T., Ozbek U. (2007). TheSOCS-1 Gene Methylation in Chronic Myeloid Leukemia Patients. Am. J. Hematol..

[91] Liu T.C., Lin S.F., Chang J.G., Yang M.Y., Hung S.Y., Chang C.S. (2003). Epigenetic Alteration of the SOCS1 Gene in Chronic Myeloid leu-Kaemia. Br. J. Haematol..

[92] Guillem V., Amat P., Cervantes F., Alvarez-Larrán A., Cervera J., Maffioli M., Bellosillo B., Collado M., Marugán I., Martínez-Ruiz F. (2012). Functional Polymorphisms in SOCS1 and PTPN22 Genes Correlate with The Response to Imatinib Treatment in Newly Diagnosed Chronic-Phase Chronic Myeloid Leukemia. Leuk. Res..

[93] Le Y., Zhu B.M., Harley B., Park S.Y., Kobayashi T., Manis J.P. (2007). SOCS3 Protein Developmentally Regulates the Chemokine Receptor CXCR4-FAK Signaling Pathway during B Lymphopoiesis. Immunity.

[94] Fierro F.A., Brenner S., Oelschlaegel U., Jacobi A., Knoth H., Ehninger G. (2009). Combining SDF-1/CXCR4 Antagonism and Chemo Therapy in Relapsed Acute Myeloid Leukemia. Leukemia.

[95] Yoshimura A., Ohkubo T., Kiguchi T., A Jenkins N., Gilbert D.J., Copeland N.G., Hara T., Miyajima A. (1995). A Novel Cytokine-Inducible Gene CIS Encodes an SH2-Containing Protein that Binds to Tyrosine-Phosphorylated Interleukin 3 and Erythropoietin Receptors. EMBO J..

[96] Li S., Chen S., Xu X., Sundstedt A., Paulsson K.M., Anderson P., Karlsson S., Sjögren H.-O., Wang P. (2000). Cytokine-Induced Src Homology 2 Protein (Cis) Promotes T Cell Receptor–Mediated Proliferation and Prolongs Survival of Activated T Cells. J. Exp. Med..

[97] Matsumoto A., Masuhara M., Mitsui K., Yokouchi M., Ohtsubo M., Misawa H., Miyajima A., Yoshimura A. (1997). CIS, A Cytokine Inducible SH2 Protein, is a Target of the JAK-STAT5 Pathway and Modulates STAT5 Activation. Blood.

[98] Zheng H., Wu X., Wu D., Jiang R.-L., Castillo E.F., Chock C.J., Zhou Q., Liu M., Dong C., Yang X.O. (2019). Treg Expression of CIS Suppresses Allergic Airway Inflammation through Antagonizing an Autonomous TH2 Program. Mucosal Immunol..

[99] Minamoto S., Ikegame K., Ueno K., Narazaki M., Naka T., Yamamoto H., Matsumoto T., Saito H., Hosoe S., Kishimoto T. (1997). Cloning and Functional Analysis of New Members of STAT Induced STAT Inhibitor (SSI) Family: SSI-2 and SSI-3. Biochem. Biophys. Res. Commun..

[100] Masuhara M., Sakamoto H., Matsumoto A., Suzuki R., Yasukawa H., Mitsui K., Wakioka T., Tanimura S., Sasaki A., Misawa H. (1997). Cloning and Characterization of Novel CIS Family Genes. Biochem. Biophys. Res. Commun..

[101] Rico-Bautista E., Flores-Morales A., Fernandez-Perez L. (2006). Suppressor of Cytokine Signaling (SOCS) 2, A Protein with Multiple Functions. Cytokine Growth Factor Rev..

[102] Dogusan Z., Hooghe-Peters E.L., Berus D., Velkeniers B., Hooghe R. (2000). Expression of SOCS Genes in Normal and Leukemic Human Leukocytes Stimulated by Prolactin, Growth Hormone and Cytokines. J. Neuroimmunol..

[103] Letellier E., Haan S. (2016). SOCS2: Physiological and Pathological Functions. Front. Biosci. (Elite Ed.).

[104] Nguyen C.H., Gluxam T., Schlerka A., Bauer K., Grandits A.M., Hackl H. (2019). SOCS2 is Part of a Highly Prognostic 4-Gene Sig-nature in AML and Promotes Disease Aggressiveness. Sci. Rep..

[105] Kazi J.U., Rönnstrand L. (2013). Suppressor of Cytokine Signaling 2 (SOCS2) Associates with FLT3 and Negatively Regulates Downstream Signaling. Mol. Oncol..

[106] Radich J.P., Dai H., Mao M., Oehler V., Schelter J., Druker B., Sawyers C., Shah N., Stock W., Willman C.L. (2006). Gene Expression Changes Associated with Progression and Response in Chronic Myeloid Leukemia. Proc. Natl. Acad. Sci. USA.

[107] Hansen N., Ågerstam H., Wahlestedt M., Landberg N., Askmyr M., Ehinger M., Rissler M., Lilljebjörn H., Johnels P., Ishiko J. (2012). SOCS2 is Dispensable for BCR/ABL1-Induced Chronic Myeloid Leukemia-Like Disease and for Normal Hematopoietic Stem Cell Function. Leukemia.

[108] Delgado-Ortega M., Melo S., Meurens F. (2011). Expression of SOCS1-7 and CIS mRNA in Porcine Tissues. Veter. Immunol. Immunopathol..

[109] Kedzierski L., Linossi E.M., Kolesnik T.B., Day E.B., Bird N.L., Kile B.T., Belz G.T., Metcalf D., Nicola N.A., Kedzierska K. (2014). Suppressor of Cytokine Signaling 4 (SOCS4) Protects against Severe Cytokine Storm and Enhances Viral Clearance during Influenza Infection. PLoS Pathog..

[110] Kobayashi D., Nomoto S., Kodera Y., Fujiwara M., Koike M., Nakayama G., Ohashi N., Nakao A. (2011). Suppressor of Cytokine Signaling 4 Detected as a Novel Gastric Cancer Suppressor Gene using Double Combination Array Analysis. World J. Surg..

[111] Xiao X., Yang D., Gong X., Mo D., Pan S., Xu J. (2018). miR-1290 Promotes Lung Adenocarcinoma Cell Proliferation and Invasion by Tar-geting SOCS4. Oncotarget.

[112] Scheitz C.J.F., Lee T.S., McDermitt D.J., Tumbar T. (2012). Defining a Tissue Stem Cell-Driven Runx1/Stat3 Signalling Axis in Epithelial Cancer. EMBO J..

[113] Brender C., Columbus R., Metcalf D., Handman E., Starr R., Huntington N., Tarlinton D., Ødum N., Nicholson S.E., Nicola N.A. (2004). SOCS5 Is Expressed in Primary B and T Lymphoid Cells but Is Dispensable for Lymphocyte Production and Function. Mol. Cell. Biol..

[114] Zhuang G., Wu X., Jiang Z., Kasman I., Yao J., Guan Y., Oeh J., Modrusan Z., Bais C., Sampath D. (2012). Tumour-Secreted miR-9 Promotes Endothelial Cell Migration and Angiogenesis by Activating the JAK-STAT Pathway. EMBO J..

[115] Hong X., Nguyen H.T., Chen Q., Zhang R., Hagman Z., Voorhoeve P.M., Cohen S.M. (2014). Opposing Activities of the R as and H Ippo Pathways Converge on Regulation of YAP Protein Turnover. EMBO J..

[116] Kang H., Sharma N.D., Nickl C.K., Devidas M., Loh M.L., Hunger S.P., Dunsmore K.P., Winter S.S., Matlawska-Wasowska K. (2018). Dysregulated Transcriptional Networks in KMT2A- and MLLT10-Rearranged T-ALL. Biomark. Res..

[117] Yuan D., Wang W., Su J., Zhang Y., Luan B., Rao H., Cheng T., Zhang W., Xiao S., Zhang M. (2018). SOCS6 Functions as a Tumor Suppressor by Inducing Apoptosis and Inhibiting Angiogenesis in Human Prostate Cancer. Curr. Cancer Drug Targets.

[118] Cheng L., Kong B.H., Zhao Y., Jiang J. (2018). miR-494 Inhibits Cervical Cancer Cell Proliferation through Upregulation of SOCS6 Ex-Pression. Oncol. Lett..

[119] Tanaka T., Arai M., Jiang X., Sugaya S., Kanda T., Fujii K., Kita K., Sugita K., Imazeki F., Miyashita T. (2014). Downregulation of microRNA-431 by Human Interferon-Beta Inhibits Viability of Medulloblastoma and Glioblastoma Cells via Upregulation of SOCS6. Int. J. Oncol..

[120] Furitsu T., Tsujimura T., Tono T., Ikeda H., Kitayama H., Koshimizu U., Sugahara H., Butterfield J.H., Ashman L.K., Kanayama Y. (1993). Identification of Mutations in the Coding Sequence of the Proto-Oncogene C-Kit in a Human Mast Cell Leukemia Cell Line Causing Ligand-Independent Activation of C-Kit Product. J. Clin. Investig..

[121] Ikeda H., Kanakura Y., Tamaki T., Kuriu A., Kitayama H., Ishikawa J., Kanayama Y., Yonezawa T., Tarui S., Griffin J.D. (1991). Expression and Functional Role of the Proto-Oncogene C-Kit in Acute Myeloblastic Leukemia Cells. Blood.

[122] Zadjali F., Pike A.C., Vesterlund M., Sun J., Wu C., Li S.S., Rönnstrand L., Knapp S., Bullock A.N., Flores-Morales A. (2011). Structural Basis for c-KIT Inhibition by the Suppressor of Cytokine Signaling 6 (SOCS6) Ubiquitin Ligase. J. Biol. Chem..

[123] Bayle J., Letard S., Frank R., Dubreuil P., De Sepulveda P. (2004). Suppressor of Cytokine Signaling 6 Associates with KIT and Regulates KIT Receptor Signaling. J. Biol. Chem..

[124] Kazi J.U., Sun J., Phung B., Zadjali F., Flores-Morales A., Rönnstrand L. (2012). Suppressor of Cytokine Signaling 6 (SOCS6) Negatively Regulates Flt3 Signal Transduction through Direct Binding to Phosphorylated Tyrosines 591 and 919 of Flt3. J. Biol. Chem..

[125] Matuoka K., Miki H., Takahashi K., Takenawa T. (1997). A Novel Ligand for an SH3 Domain of the Adaptor Protein Nck Bears an SH2 Domain and Nuclear Signaling Motifs. Biochem. Biophys. Res. Commun..

[126] Krebs D.L., Uren R., Metcalf D., Rakar S., Zhang J.-G., Starr R., DE Souza D., Hanzinikolas K., Eyles J., Connolly L.M. (2002). SOCS-6 Binds to Insulin Receptor Substrate 4, and Mice Lacking the SOCS-6 Gene Exhibit Mild Growth Retardation. Mol. Cell. Biol..

[127] Martens N., Uzan G., Wery M., Hooghe R., Hooghe-Peters E.L., Gertler A. (2005). Suppressor of Cytokine Signaling 7 Inhibits Prolactin, Growth Hormone, and Leptin Signaling by Interacting with STAT5 or STAT3 and Attenuating Their Nuclear Translocation. J. Biol. Chem..

[128] Ge D., Gao A.C., Zhang Q., Liu S., Xue Y., You Z. (2011). LNCaP Prostate Cancer Cells with Autocrine Interleukin-6 Expression are Resistant to IL-6-Induced Neuroendocrine Differentiation due to Increased Expression of Suppressors of Cytokine Signaling. Prostate.

[129] Liu X.H., Xu S.B., Yuan J., Li B.H., Zhang Y., Yuan Q. (2009). Defective Interleukin-4/Stat6 Activity Correlates with Increased consti-tutive Expression of Negative Regulators SOCS-3, SOCS-7, and CISH in Colon Cancer Cells. J. Interferon Cytokine Res..

[130] Sutherland K.D., Lindeman G.J., Choong D.Y.H., Wittlin S., Brentzell L., Phillips W., Campbell I.G., E Visvader J. (2004). Differential Hypermethylation of SOCS Genes in Ovarian and Breast Carcinomas. Oncogene.

[131] Yoshikawa H., Matsubara K., Qian G.-S., E Jackson P., Groopman J.D., Manning J.E., Harris C.C., Herman J.G. (2001). SOCS-1, a Negative Regulator of the JAK/STAT Pathway, is Silenced by Methylation in Human Hepatocellular Carcinoma and Shows Growth-Suppression Activity. Nat. Genet..

[132] Fukushima N., Sato N., Sahin F., Su G., Hruban R.H., Goggins M. (2003). Aberrant Methylation of Suppressor of Cytokine Signalling-1 (SOCS-1) Gene in Pancreatic Ductal Neoplasms. Br. J. Cancer.

[133] Kang X.-C., Chen M.-L., Yang F., Gao B.-Q., Yang Q.-H., Zheng W.-W., Hao S. (2016). Promoter Methylation and Expression of SOCS-1 Affect Clinical Outcome and Epithelial-Mesenchymal Transition in Colorectal Cancer. Biomed. Pharmacother..

[134] Fukui H., Watari J., Zhang X.X., Ran Y., Tomita T., Oshima T., Hirota S., Miwa H. (2020). Phosphorylated STAT3 Expression linked to SOCS3 Methylation is Associated with Proliferative Ability of Gastric Mucosa in Patients with Early Gastric Cancer. Oncol. Lett..

[135] Letellier E., Schmitz M., Baig K., Beaume N., Schwartz C., Frasquilho S., Antunes L., Marcon N., Nazarov P., Vallar L. (2014). Identification of SOCS2 and SOCS6 as Biomarkers in Human Colorectal Cancer. Br. J. Cancer.

[136] Liu S., Ren S., Howell P., Fodstad O., Riker A.I. (2008). Identification of Novel Epigenetically Modified Genes in Human Melanoma via Promoter Methylation Gene Profiling. Pigment. Cell Melanoma Res..

[137] Fandy T.E., Herman J.G., Kerns P., Jiemjit A., Sugar E.A., Choi S.-H., Yang A.S., Aucott T., Dauses T., Odchimar-Reissig R. (2009). Early Epigenetic Changes and DNA Damage do not Predict Clinical Response in an Overlapping Schedule of 5-Azacytidine and Entinostat in Patients with Myeloid Malignancies. Blood.

[138] Wu Q.-Y., Zhu Y.-Y., Liu Y., Wei F., Tong Y.-X., Cao J., Zhou P., Niu M.-S., Li Z.-Y., Zeng L.-Y. (2018). CUEDC2, A Novel Interacting Partner of the SOCS1 Protein, Plays Important Roles in the Leukaemogenesis of Acute Myeloid Leukaemia. Cell Death Dis..

[139] Saudemont A., Hamrouni A., Marchetti P., Liu J., Jouy N., Hetuin D., Colucci F., Quesnel B. (2007). Dormant Tumor Cells Develop Cross-Resistance to Apoptosis Induced by CTLs or Imatinib Mesylate via Methylation of Suppressor of Cytokine Signaling 1. Cancer Res..

[140] Pena M.C.R., Pardini M.I.M.C., Colturato V.A.R., Pinheiro N.A. (2009). Methylation Status of the SOCS 1 and JUNB Genes in Chronic Myeloid Leukemia Patients. Rev. Bras. De Hematol. E Hemoter..

[141] Elias M.H., Azlan H., Baba A.A., Ankathil R. (2018). Aberrant DNA Methylation of SOCS1 Gene is Not Associated with Resistance to Imatinib Mesylate among Chronic Myeloid Leukemia Patients. Cardiovasc. Hematol. Disord. Targets.

[142] Naser S.A. (2020). MiR-146a rs2910164 G> C Polymorphism Modulates Notch-1/IL-6 Signaling During Infection: A Possible Risk Factor for CROHN’S Disease. Gut Pathogens.

[143] Mendell J.T., Olson E.N. (2012). MicroRNAs in Stress Signaling and Human Disease. Cell.

[144] Mardani R., Jafari Najaf Abadi M.H., Motieian M., Taghizadeh-Boroujeni S., Bayat A., Farsinezhad A., Hayat S.M.G., Motieian M., Pourghadamyari H. (2019). MicroRNA in Leukemia: Tumor Suppressors and Oncogenes with Prognostic Potential. J. Cell. Physiol..

[145] Eis P., Tam W., Sun L., Chadburn A., Li Z., Gomez M.F., Lund E., Dahlberg J.E. (2005). Accumulation of miR-155 and BIC RNA in Human B Cell Lymphomas. Proc. Natl. Acad. Sci. USA.

[146] Cammarata G., Augugliaro L., Salemi D., Agueli C., La Rosa M., Dagnino L., Civiletto G., Messana F., Marfia A., Bica M.G. (2010). Differential Expression of Specific microRNA and their Targets in Acute Myeloid Leukemia. Am. J. Hematol..

[147] Mattiske S., Suetani R.J., Neilsen P., Callen D. (2012). The Oncogenic Role of miR-155 in Breast Cancer. Cancer Epidemiol. Biomark. Prev..

[148] Yang M., Shen H., Qiu C., Ni Y., Wang L., Dong W. (2013). High Expression of miR-21 and miR-155 Predicts Recurrence and Un-Favourable Survival in Non-Small Cell Lung Cancer. Eur. J. Cancer.

[149] Wu T., Xie M., Wang X., Jiang X., Li J., Huang H. (2012). miR-155 Modulates TNF-α-Inhibited Osteogenic Differentiation by Targeting SOCS1 Expression. Bone.

[150] Yao R., Ma Y.-L., Liang W., Li H.-H., Ma Z.-J., Yu X., Liao Y.-H. (2012). MicroRNA-155 Modulates Treg and Th17 Cells Differentiation and Th17 Cell Function by Targeting SOCS1. PLoS ONE.

[151] O’Connell R.M., Rao D., Chaudhuri A.A., Boldin M., Taganov K.D., Nicoll J., Paquette R.L., Baltimore D. (2008). Sustained Expression of microRNA-155 in Hematopoietic Stem Cells Causes a Myeloproliferative Disorder. J. Exp. Med..

[152] Babar I.A., Cheng C.J., Booth C.J., Liang X., Weidhaas J.B., Saltzman W.M. (2012). Nanoparticle-based Therapy in an In Vivo mi-croRNA-155 (miR-155)-Dependent Mouse Model of Lymphoma. Proc. Natl. Acad. Sci. USA.

[153] Di Iasio M.G., Norcio A., Melloni E., Zauli G. (2012). SOCS1 is Significantly Up-Regulated in Nutlin-3-Treated p53 Wild-Type B Chronic Lymphocytic Leukemia (B-CLL) Samples and Shows an Inverse Correlation with miR-155. Investig. New Drugs.

[154] Mignacca L., Saint-Germain E., Benoit A., Bourdeau V., Moro A., Ferbeyre G. (2016). Sponges Against miR-19 and miR-155 Reactivate the p53-Socs1 Axis in Hematopoietic Cancers. Cytokine.

[155] Zanette D.L., Rivadavia F., Molfetta G.A., Barbuzano F.G., Proto-Siqueira R., Silva W.A. (2007). miRNA Expression Profiles in Chronic Lymphocytic and Acute Lymphocytic Leukemia. Braz J. Med. Biol Res..

[156] Qin S., Ai F., Ji W.-F., Rao W., Zhang H.-C., Yao W.-J. (2013). miR-19a Promotes Cell Growth and Tumorigenesis through Targeting SOCS1 in Gastric Cancer. Asian Pac. J. Cancer Prev..

[157] Wang X.G., Chen Z. (2015). MicroRNA-19a Functions as an Oncogenic microRNA in Non-Small Cell Lung Cancer by Targeting the Sup-pressor of Cytokine Signaling 1 and Mediating STAT3 Activation. Int. J. Mol. Med..

[158] Pichiorri F., Suh S.-S., Ladetto M., Kuehl M., Palumbo T., Drandi D., Taccioli C., Zanesi N., Alder H., Hagan J. (2008). MicroRNAs Regulate Critical Genes Associated with Multiple Myeloma Pathogenesis. Proc. Natl. Acad. Sci. USA.

[159] Collins A.S., McCoy C., Lloyd A.T., O’Farrelly C., Stevenson N.J. (2013). miR-19a: An Effective Regulator of SOCS3 and Enhancer of JAK-STAT Signalling. PLoS ONE.

[160] Lerut E., Gessler M., Schubert M., Kalogirou C., Kneitz S., Kneitz B., Van Poppel H., Riedmiller H., Scholz C.J., Spahn M. (2014). Survival in Patients with High-Risk Prostate Cancer is Predicted by miR-221, Which Regulates Proliferation, Apoptosis, and Invasion of Prostate Cancer Cells by Inhibiting IRF2 and SOCS3. Cancer Res..

[161] Zhang J.W., Wang X., Li G.C., Wang D., Han S., Zhang Y.D., Luo C.H., Wang H.W., Jiang W.J., Li C.X. (2020). MiR-30a-5p Promotes Cholangiocarcinoma Cell Proliferation through Targeting SOCS3. J. Cancer.

[162] Liu Y.-X., Wang L., Liu W.-J., Zhang H.-T., Xue J.-H., Zhang Z.-W., Gao C.-J. (2016). MiR-124-3p/B4GALT1 Axis Plays an Important Role in SOCS3-Regulated Growth and Chemo-Sensitivity of CML. J. Hematol. Oncol..

[163] Miao F., Zhu J., Chen Y., Tang N., Wang X., Li X. (2015). MicroRNA-183-5p Promotes the Proliferation, Invasion and Metastasis of Human Pancreatic Adenocarcinoma Cells. Oncol. Lett..

[164] Sha C., Jia G., Jingjing Z., Yapeng H., Zhi L., Guanghui X. (2020). miR-486 is Involved in the Pathogenesis of Acute Myeloid Leukemia by Regulating JAK-STAT Signaling. Naunyn-Schmiedeberg’s Arch. Pharmacol..

[165] Liu H.-M., Guo C.-L., Zhang Y.-F., Chen J.-F., Liang Z.-P., Yang L.-H. (2021). Leonurine-Repressed miR-18a-5p/SOCS5/JAK2/STAT3 Axis Activity Disrupts CML Malignancy. Front. Pharmacol..

[166] Zhou X., Xia Y., Li L., Zhang G. (2014). MiR-101 Inhibits Cell Growth and Tumorigenesis of Helicobacter Pylori Related Gastric Cancer by Repression of SOCS2. Cancer Biol. Ther..

